# EDI200 therapeutic trial

**DOI:** 10.1186/1746-160X-8-S1-I18

**Published:** 2012-05-25

**Authors:** Kenneth Huttner

**Affiliations:** 1 Edimer Pharmaceuticals, Cambridge, USA

## 

X-linked hypohidrotic ectodermal dysplasia (XLHED) is the most common of the ectodermal dysplasias, presenting with hypohidrosis, hypodontia, hypotrichosis and mucociliary gland hypoplasia. As an X-linked disorder, the phenotype is more consistently severe in affected males than in affected females and is associated with a risk for serious and potentially life-threatening hyperthermia and respiratory infections in early childhood.

Although XLHED has been described in publications since the mid 19^th^ century, it was not until the genomics revolution that the underlying cause was identified as a deficiency of the ectodermal morphogen ectodysplasin. Over 200 mutations of the corresponding gene, EDA, have been described that produce a deficiency in the activity of its primary translational product EDA-A1. As a key regulator of the initiation, maturation and maintenance of ectodermal appendages, the exact timing for EDA-A1 activity in development may vary both between tissues and between species. This was demonstrated in two animal models of XLHED, the mouse and the dog, where a recombinant form of EDA-A1 (EDI200) was administered either antenatally or postnatally. Perhaps surprisingly, in the dog model most analogous to human XLHED, postnatal EDI200 administration was associated with a sustained, reproducible and clinically meaningful correction of the XLHED phenotype.

Detailed studies of the response to EDI200 in both animal models have provided a roadmap for clinical trials involving EDI200 as an EDA-A1 replacement in patients with XLHED. Under consideration for 2012 is the initiation of studies in a small number of XLHED-affected adults and newborns to confirm the safety of EDI200 in these populations and to assess the potential for ameliorating the clinical signs and symptoms. Key aspects of this clinical development plan will be discussed including selection of dose and dosing regimen, the window of efficacy and options for bioactivity monitoring.

